# Application of Plant Growth Promoting Rhizobacteria to Improve Soil Chemical and Biological Properties and Its Effect on Growth, Physiology and Yield of Okra (*Abelmoschus esculentus* L.)

**DOI:** 10.21315/tlsr2025.36.2.4

**Published:** 2025-07-31

**Authors:** Zakiah Mustapha, Nik Nurnaeimah Nik Mohamad Nasir, Mohd Khairi Che Lah, Norhayati Ngah, Khamsah Suryati Mohd, Radziah Othman, Hafizan Juahir

**Affiliations:** 1 School of Agriculture Science and Biotechnology, Faculty of Bioresources and Food Industry, Universiti Sultan Zainal Abidin, Besut Campus, 22200 Besut, Terengganu, Malaysia; 2 Faculty of Plantation and Agrotechnology, Universiti Teknologi MARA, 26400 Jengka, Pahang, Malaysia; 3 Department of Land Management, Faculty of Agriculture, Universiti Putra Malaysia, 43400 Serdang, Selangor, Malaysia

**Keywords:** Biofertiliser, BRIS Soil, Chemical Fertiliser, Microbial Consortium, PGPR, Baja Biologi, Tanah BRIS, Baja Kimia, Konsortium Mikrob, PGPR

## Abstract

The use of Plant Growth-Promoting Rhizobacteria (PGPR) as a biofertiliser was proven to be successful in the optimisation of plant growth and yield. A field experiment was conducted to evaluate the effectiveness of the PGPR on okra growth, physiology, yield and soil physicochemical properties. The okra was planted and fertilised with organic material (goat dung) at 500 g/plant, NPK fertiliser at 100 g/plant (T1) and 70 g/plant for T2, T3, T4 and T5, respectively. The BRIS soil isolated PGPR, namely UA 1 (*Paraburkholderia unamae*), UA 6 (*Bacillus amyloliquefaciens*) and UAA 2 (*Enterobacter asburiae*) propagated in 6% molasses medium were inoculated with the amount of 40 mL for single strain treatment (T2–T4) and 15 mL of each bacterial inoculum for mixed strains treatment (T5). Results showed that inoculation with PGPR in single or mixed strains has significantly decreased the use of 30% NPK fertiliser and promoted okra growth, physiology, yield and soil chemical properties and bacterial count. Mixed strains (T5) have significantly shown the highest performance with increments of 27.85% of leaf number, 28.56% of okra number, 27.90% of yield per plant and 25.83% of plant total dry biomass. The plant net photosynthesis treated with mixed strains also recorded a 5.27% increment with 26.96% of nitrogen content and 22.79% bacteria count in the soil. The findings of this study suggested that the BRIS soil PGPR inoculants may reduce the amount of chemical fertiliser and have a significant potential to be used as biofertiliser in sustainable agriculture to increase plant growth and yield and soil fertility.

## Abstrak

Penggunaan bakteria rizobakteria penggalak pertumbuhan tanaman (PGPR) sebagai baja biologi telah terbukti berjaya dalam pengoptimuman pertumbuhan dan hasil tanaman. Satu eksperimen lapangan telah dijalankan untuk menilai keberkesanan PGPR terhadap pertumbuhan, fisiologi, hasil dan sifat fiziko-kimia tanah okra. Okra ditanam dan dibaja dengan bahan organik (najis kambing) sebanyak 500 g/tumbuhan, baja NPK masingmasing sebanyak 100 g/tumbuhan (T1) dan 70 g/tumbuhan untuk T2, T3, T4 dan T5. PGPR yang diasingkan dari tanah BRIS, iaitu UA 1 (*Paraburkholderia unamae*), UA 6 (*Bacillus amyloliquefaciens*) dan UAA 2 (*Enterobacter asburiae*) yang dibiakkan dalam medium molases 6% diinokulasi dengan sebanyak 40 mL untuk rawatan strain tunggal (T2–T4) dan 15 mL bagi setiap inokulum bakteria untuk rawatan strain campuran (T5). Keputusan menunjukkan bahawa inokulasi dengan PGPR dalam strain tunggal atau campuran telah mengurangkan secara signifikan penggunaan 30% baja NPK dan mempromosikan pertumbuhan, fisiologi, hasil dan sifat kimia tanah serta jumlah bakteria. Strain campuran (T5) telah menunjukkan prestasi tertinggi dengan peningkatan 27.85% dalam bilangan daun, 28.56% dan 27.90% dalam bilangan dan hasil okra setiap pokok dan 25.83% dalam jumlah bahan kering keseluruhan pokok. Fotosintesis bersih pokok yang dirawat dengan strain campuran juga mencatat peningkatan 5.27% dengan kandungan nitrogen sebanyak 26.96% dan jumlah bakteria dalam tanah sebanyak 22.79%. Penemuan kajian ini mencadangkan bahawa inokulan PGPR tanah BRIS mungkin mengurangkan jumlah baja kimia dan mempunyai potensi yang signifikan untuk digunakan sebagai baja biologi dalam pertanian lestari untuk meningkatkan pertumbuhan pokok, hasil, dan kesuburan tanah.

**Highlights**

- A field experiment inoculation of the single and mixed strains of BRIS soil PGPR on okra.
- BRIS soil PGPR has significantly promoted okra growth, physiology and yield, soil chemical properties and soil bacterial count.
- Inoculation of BRIS soil PGPR can reduce the use of chemical fertiliser by 30%.

## INTRODUCTION

The increasing demand for high-yield and quality agricultural products causes an intensive farming practice leading to the use of extensive chemical inputs that are costly and may create environmental and health problems. The amount of organic matter content in agricultural soil has also declined because of the continuous use of chemical fertilisers. This situation led to the depletion of beneficial microorganisms which in turn reduced the soil productivity (Baliah & Muthulakshmi 2017). Since the use of chemical inputs has many negative impacts in the long term, adding more minerals or plant nutrients should be part of the plan to increase agricultural output. It could be achieved through an alternative approach that is more environmentally friendly such as the use of effective microbes or plant growth-promoting rhizobacteria (PGPR) as biofertiliser.

Biofertiliser has gained interest recently since it can minimise the amount of chemical inputs used while increasing output. One of the most crucial elements for biological management, promoting the growth of various plant parts and fostering sustainable agriculture, is the diversity of microbes in the soil (Abdelmoaty *et al*. 2021). Numerous researchers have studied plant growth improvement with single or combined inoculation of symbiotic and non-symbiotic microorganisms. Additionally, reports have demonstrated the effective, minimal substitution of conventional fertiliser with biofertiliser at a reduced cost. Several PGPRs were already well-recognised for the emergence of seeds, enhanced plant growth, improved crop production and used as biofertiliser in agriculture development (Kumari *et al*. 2019).

The PGPR mode of action differs and can be effectively used either singly or in combination. However, many researchers have reported inconsistent results on the effects of bacterial inoculation on crops in the laboratory, glasshouse or field (Akhtar & Siddiqui 2010; Gouda *et al*. 2018). Thus, the successful colonisation and beneficial effects of PGPR under lab or greenhouse conditions may not be the same as in the field. This might be due to several factors such as the rate and timing of bacterial application and other environmental factors such as soil condition and population of other indigenous microbes. Therefore, a field trial is crucial in a PGPR assessment to ensure the effectiveness and successful inoculation on plant. Evaluation is required for field, greenhouse or glasshouse experiments that determine the effects of PGPR in conjunction with traditional methods. It includes the use of organic materials and other chemical inputs while maintaining the homogeneity of external factors such as soil conditions, water and light.

Beach Ridges Interspersed with Swales soil or known as BRIS soil is the problematic sandy soil with more than 90% sand situated in the sandy beach ridges of the Kelantan-Terengganu Plains in Malaysia. This type of soil is regarded as problematic lowland soil for agriculture because of its very sandy texture with low fertility, high temperature, low cation exchange capacity and low water holding capacity. It also has an uncertain condition where there is a monsoon season with high rainfall that sometimes inundates this area causing relatively lower temperatures and higher humidity than its usual condition. However, there are few species of plants recorded to be dominant in this area at Besut, Terengganu such as the Acacia tree (*Acacia mangium*) and Gelam tree (*Melaleuca cajuputi* Powell). BRIS soil is also known as marine soil (Department of Agriculture Malaysia [DOA] 2023) which is the dominant coastal area in the eastern part of peninsular Malaysia. Nevertheless, BRIS soil is not located in this area only. This type of soil can also be found in several types of coastal sandy soil areas in the world like Andhra Pradesh (India), and the coastal central provinces of Vietnam (Hoang *et al.* 2010; Ishaq *et al.* 2014).

Application of PGPR that was isolated from sandy soil gives an interesting result on the growth performance of crops. The PGPR can significantly increase the plant physiology, biochemical content, improve soil fertility and yield of the plant even the crops grown in harsh environments (Khan *et al.* 2019). Successful isolation of PGPR from BRIS soil gives a new opportunity and alternative for their beneficial application on plants in other types of soil, especially the sandy soil. PGPRs that were used in this study are believed to be hardy and could be used in the local area or other places with the same or different soil and environment characteristics. In the previous study by Mustapha *et al.* (2018), three types of PGPR namely UA 1 (*Paraburkholderia unamae*), UA 6 (*Bacillus amyloliquefaciens*) and UAA 2 (*Enterobacter asburiae*) have been previously isolated from the rhizosphere of *Acacia mangium* that grow in the BRIS soil area.

The isolated PGPRs by Mustapha *et al.* (2018) have been proven in the laboratory to have plant growth-promoting (PGP) characteristics such as the ability to fix atmospheric nitrogen, solubilise phosphate and potassium, producing IAA and siderophores. However, the performance of the isolated PGPR was never been tested outside the laboratory, on plants in the field area. In this study, okra (*Abelmoschus esculentus* L.) was selected as the test plant since it is the most common and wholesome vegetable grown worldwide due to its taste and nutritious qualities. Moreover, this plant is easy to grow, hardy and less affected by pests and diseases, thus suitable as the initial test crop in this field study. In field conditions, the effects of isolated PGPR in single and mixed strains on plant growth, physiology and yield were determined. Also evaluated were the effects of bacterial inoculations on the chemical composition of the soil and the number of bacteria present.

## MATERIALS AND METHODS

### Preparation of Bacterial Inoculum

Three types of PGPR were isolated from the rhizosphere of *Acacia mangium* at BRIS soil in Tembila Besut Terengganu and identified as *Paraburkholderia unamae* (UA 1). *Bacillus amyloliquefaciens* (UA 6) and *Enterobacter asburiae* (UAA 2) were grown in 6% molasses medium for three days and kept at 4**°**C in the chiller for further use. The optical density (OD) of the cell suspension was adjusted to 0.4 A at 600 nm using UV-VIS spectrophotometer (approximately 3–4 **×** 10^7^ cells/mL).

### Study Site

The field experiment was undertaken at the Faculty of Bioresources and Food Industry (FBIM) farm (5°45’36.5”N; 102°37’18.0”E), Universiti Sultan Zainal Abidin, Besut Campus, Terengganu, Malaysia. The field experiment took 78 days from April to June in tropical weather at average temperature ranges between 30**°**C to 40**°**C .

### Experimental Design

The experiment was laid out in Randomised Complete Block Design (RCBD) consisting of four blocks with four replications and five treatments. The treatment was labelled as T1, T2, T3, T4 and T5. T1 was the control and T2–T5 were treated with the selected BRIS soil PGPR. An amount of 40 mL for single strain (UA 1/UA 6/UAA 2) treatment (T2–T4) and 15 mL each or mixed strains (UA 1 + UA 6 + UAA 2) treatment (T5) of each bacterial inoculum in 6% molasses medium was poured into the soil in each pot of okra plant. Inoculation was done at 1 week after sowing and repeated at weeks 3, 5, 7 and 9.

The okra seeds (Leckat Seeds No. 979) were germinated under a shaded area in a plastic seed germination tray with a size of 3.7 cm **×** 4 cm. Germination media (Superb Agromedia™) was used to germinate the okra seeds. Germinated seeds were transferred into the polybag after two weeks of germination to be grown in the open field condition.

The size of the polybag that has been used was 30 cm **×** 45 cm. The soil mixture was prepared at a ratio of 3:1 (top soil:sand). An amount of 14.5 kg soil mixture was added and mixed with 500 g organic material (chicken manure) per polybag. The polybags were organised at a spacing of 90 cm in the row and 120 cm between rows in the open field. The NPK Fertiliser (Nitrophoska® Blue fertiliser, Behn Meyer) doses used in this study were 70 g/plant except for T1 which used 100 g/plant (Table 1). NPK fertiliser was given standardly for all treatments at weeks 2, 4, 6 and 8 after sowing. Each plant in T1 was given 10 g, 20 g, 30 g and 40 g NPK fertiliser while T2–T5 were given with 10 g, 10 g, 20 g and 30 g for the respective weeks. Watering was done manually on a daily basis at approximately 3 L for each plant. Weeding was carried out manually as frequently as the weeds emerged.

### Measurement of Okra Growth, Physiology and Yield Parameters

Growth parameters (plant height, stem diameter, number and size of leaves) and physiological parameters (net photosynthesis, stomata conductance, internal sub-stomata CO_2_, transpiration rate and leaf-to-air vapour pressure deficit) were recorded at day 60 after sowing. The leaf size was measured using LI-3100C Area Meter while the physiological parameters were measured using LI-COR Portable Photosynthesis and Fluorescence System (LI-6400XT). Fruit production of okra was manually counted, harvested, weighed and recorded. The process started on day 50 and repeated every four-day intervals until day 78. On day 78, the okra plant was harvested for further study. The leaves and stem of okra were harvested and the soil from the polybag pot was washed away to collect the root samples. The root samples were thoroughly washed with tap water and blotted dried with tissue papers before sun drying. All leaves, stems and roots of the okra were then placed in an oven (Protech FDD-1000) at 70**°**C for 72 h until a constant weight was achieved. The dried weight was recorded for every treatment and the total dry biomass was calculated.

### Soil Analysis

The soil samples were collected from each treatment after harvesting (day 78) at 15 cm depth. It was air-dried for 48 h and sieved through a 2.0 mm sieve before being mixed into a single composite sample and kept in the 4**°**C chiller for further soil analysis. The soil pH was analysed using the electrometric method and cations exchange capacity (CEC) used the modified BaCl_2_ method. The total organic carbon and sulfur were determined by the dry combustion method while total N was determined by the Kjeldahl method. Determination of available phosphorus and potassium used aqua regia digestion method and analysed using ICP–OES instrument (Optima 2000DV, Perkin Elmer). Finally, the total bacteria count was done using the spread plate method on Nutrient Agar (NA) medium.

### Statistical Analysis

The data of okra growth, physiology and yield parameters were subjected to a two-way analysis of variance (ANOVA) and the data of soil parameters and bacterial total count were subjected to one-way ANOVA from SPSS version 21. Multiple comparisons between treatment means were done using Tukey’s Test. The differences at *P* < 0.05 were considered significant.

## RESULTS

### Growth Parameters of Okra

The results showed that there was a difference in okra growth as affected by the application of 100% (T1) or 70% NPK fertiliser with PGPR treatment (T2–T5). Significance differences were recorded on the leaf development which is the number and size of the leaf. Meanwhile, the increased results for stem size and plant height were not significant (Table 1). Treatment with mixed strains culture of UA 1, UA 6 and UAA 2 (T5) had the highest leaf number with a mean average of 38.75 leaves per plant. The results were followed by a single strain inoculation of UA 6 (T3) with 36.44 leaves per plant. A single strain of UA 1 (T2) had 35.13 leaves per plant and the lowest UAA 2 (T4) had 34.75 leaves per plant. Control treatment with only NPK fertiliser without any bacterial application (T1) had the lowest leaf numbers with an average of 30.31 leaves per plant. In terms of leaf size, T1 only had an average area of 692.20 cm^2^/leaf while treatment with PGPR mix strains gave an average of up to 711.40 cm^2^/leaf.

### Physiological Parameters of Okra

Treatment with PGPR (T2–T5) does give a difference in okra’s physiology but the results were not significant to T1 (Table 2). However, the increment of transpiration rate was significantly higher in bacterial application compared to control. The highest transpiration rate was recorded by T2 and this treatment also showed significantly higher stomata conductance compared to the control (T1).

### Yield of Okra

**Figure 1:**
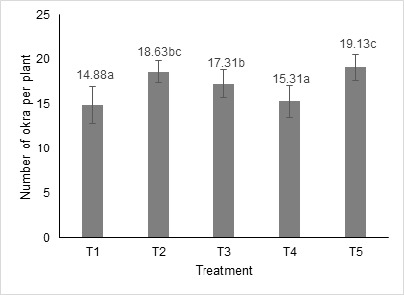
Effect of BRIS soil PGPR on the mean number of okra fruit per plant. Mean ± standard deviation (error bars) with the same letters are not significantly different at *P* < 0.05 Tukey’s multiple comparison.

**Figure 2:**
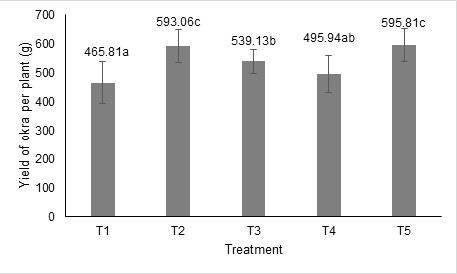
Effect of BRIS soil PGPR on the okra yield (fruit weight) per plant (g). Means ± standard deviation (error bars) with the same letters are not significantly different at *P* < 0.05 Tukey’s multiple comparison.

Eight times harvesting starting at day 50 after sowing until day 78 showed that the average number of okra fruit and yield (total fruit weight) per plant was increased with BRIS soil bacterial treatment (Figs. 1 and 2). Treatment T2, T3 and T5 showed a significant increase (*p* < 0.05) compared to T1. Treatment with a single strain of UA 1 (T2) and mixed strains of bacterial culture (T5) produced the highest number and yield of okra fruit per plant. The number of okra fruit increments was between 25.20% to 28.56% and yield increments between 27.32% to 27.91%, respectively, for T2 and T5. The strain UA 6 also produced significantly higher okra fruit number (16.33%) and yield (15.74%) compared to control. Overall, mixed strains of bacterial treatment produced the highest number and yield of okra fruit per plant, followed by single-strain bacterial treatment of UA1, UA 6 and UAA 2.

### Dry Biomass of Okra

It was found that there was an increment in the dry weight of leaves and stems of plants treated with PGPR but the results were not significant to control. The root’s dry biomass and total dry biomass showed a significant increase with bacterial treatment compared to the control (Table 3). However, treatment with UA 1 (T2) and mixed strains (T5) showed a significantly higher root dry weight and total dry biomass compared to control (T1). While treatment with UA 6 (T3) significantly increased plant total dry biomass compared to control (T1). The treatment T2, and T5 showed a significant increment between 24.77% to 38.79% in dry root biomass and 19.48% to 25.83% in total dry biomass. Overall, mixed strains showed the highest increment in dry biomass of different plant parts and total dry biomass per plant followed by UA 1, UA 6 and UAA 2 (Table 3).

### Soil Chemical Content and Bacterial Count

Table 4 represents the okra soil properties with different treatments (T1–T5) at 78 days after sowing. Treatment with PGPR inoculant into the soil (T2–T5) has significantly increased the number of total bacterial count compared to no addition of bacteria (T1). The soil nitrogen, phosphorus and potassium were also increased as the soil was treated with PGPR compared to the untreated soil. There was also a slight increase in soil pH and CEC value with the addition of bacteria into the soil. Overall, treatment with UA 1 (T2) has shown good performance in the field with the highest significant value of CEC (7.123 Cmol_c_/kg), soluble phosphorus (27.870 mg/kg) and potassium (37.863 mg/kg) in the soil followed by mixed PGPR strains (T5). Treatment with UA 6 (T3) showed the highest nitrogen value in the soil (58.707 mg/kg). In contrast, treatment with UAA 2 (T4) showed the lowest effect on soil properties compared to other selected BRIS soil bacterial treatments.

## DISCUSSION

In this study, the strain UA 1 and UA 6 showed a better effect on okra compared to the strain UAA 2. While the use of these three strains in a consortium (mix form) showed better performance on okra compared to the use of a single strain alone. A lot of studies by other researchers have proven that inoculation with PGPR does increase plant growth, development and yield in many crops and plants. Nevertheless, it was also effective on many legumes such as maize (Falik *et al.* 1989) and non-legume plants such as potato and sorghum (Gaskins *et al.* 1985; Sarig *et al.* 1988).

The results from this study were important as they proved that the use of BRIS soil PGPR treatment either in single or mixed form could enhance plant growth and yield. At the same time, an amount of 30% chemical NPK fertiliser could be reduced which is important for sustainable agriculture. The finding in this study is almost similar to Mia *et al.* (2009) who discovered UPMB10 (*Bacillus subtilis*) and *Azospirillum* Sp7 inoculation combined with a 33% fertiliser-N supply effectively produced an equivalent total dry matter of banana plants as those provided with 100% fertiliser-N. It was also in agreement with Rafique *et al.* (2018) that the growth parameters of okra such as leaf number, plant height, root length and dry biomass have significantly increased by the PGPR treatment. In addition, as reported by Baliah and Muthulakshmi (2017), the application of PGPR-enriched vermicompost has a significant effect on the okra growth, yield, nutritive value such as ascorbic acid and glucose content and biochemical characteristics such as total chlorophyll and protein content.

Thus, the PGPR inoculation in regular application has managed to decrease the use of chemical fertiliser to get a higher yield than the use of standard fertiliser rate without bacterial inoculants. The use of organic material together with beneficial microorganisms in the proper and regular way has been discussed by many researchers could reduce the use of chemical fertiliser to 30%, 50%, 70% or even 100% to achieve the desired yield as by using chemical fertiliser. It was also discovered that applying PGPR strains in combination or as a consortium could improve the growth response. It is in agreement with several previous studies on the advantages of PGPR consortium application to increase and boost plant growth and yield (Karthikeyan *et al*. 2010; Widawati & Suliasih 2018).

The results from this study also proved that plant physiology and growth can be enhanced by PGPR inoculation. However, the growth responses are varied between the uses of different rhizobacterial strains. In the study of maize by Kifle and Laing (2016), they found that the variation in growth and yield parameters could be attributed to the variation in the rate of physiology characteristics such as stomata conductance and chlorophyll content by the effect of PGPR inoculation from the genus *Paraburkholderia, Bacillus, Enterobacter, Pseudomonas* and *Pantoea*. The result was also recorded by Knoth *et al.* (2014) that the biomass and rate of net CO_2_ assimilation were higher in maize plants inoculated with *Paraburkholderia vietnamienis* compared to the uninoculated plants. These reasons might be correlated to the findings in our study.

The PGPR strains used in this study were identified as *Paraburkholderia unamae* (UA 1), *Bacillus amyloliquefaciens* (UA 6) and *Enterobacter asburiae* (UAA 2) which have long been reported to be beneficial to various crops (Mustapha *et al*. 2022). Through several different mechanisms, PGPR can protect plants from abiotic stress and disease while also promoting plant development (Mohammad *et al*. 2021). Several important bacterial characteristics, such as biological nitrogen fixation (BNF), phosphate and potassium solubilisation, production of siderophores and phytohormones and 1-Aminocyclopropane-1-carboxylate (ACC) deaminase activity, can be assessed as PGP traits. The agronomic practice efficiency could be enhanced by successful bacterial inoculations with efficient PGP characteristics. As a result, it could also reduce production costs and environmental pollution while reducing or eliminating the use of chemical fertilisers.

The isolated BRIS soil PGPR in this study also has multiple beneficial PGP characteristics including the ability to fix nitrogen (Mustapha *et al*. 2018). BNF by bacteria was proven to have a direct and strong relationship with nitrogen uptake from soil. In this study, the use of BRIS soil PGPR has significantly increased the size and number of leaves. This situation will increase the photosynthesis rate, thus improving plant growth and development. Moreover, nitrogen availability could also improve root length and dry weight, thus improving dry matter accumulation and partitioning into the economic part of a plant (Mastrodomenico & Purcell 2012).

PGPR infection and establishing a symbiotic relationship with the plant’s roots and augmented plant growth have long been reported. According to Salantur *et al.* (2006), the application of bacterial inoculum has been reported to increase the growth and yield of many plants such as rice, wheat and maize through nitrogen fixation, solubilisation of phosphorus and production of plant growth regulators. PGP hormones like IAA and GA_3_, released by *Azotobacter* and *Rhizobium* strains have also improved the okra’s growth (Rafique *et al.* 2018). According to Li *et al.* (2009), plant biomass production is positively related to plant development such as plant height, leaf area, root weight and photosynthetic ability. There is a positive relationship between plant growth such as height and total leaf area with biomass production (Hossain *et al*. 2011).

Plant growth, health, production and soil fertility are all determined by interactions between microbes and plants in the rhizosphere. Numerous aspects affect the effectiveness and efficiency of PGPR as inoculants for crops, one of which is the bacteria’s capacity to colonise plant roots and improve soil health (de Souza *et al*. 2015). A high bacterial population is needed in a successful plant-microbe interaction. In the soil or root environment, the PGPR must survive in competition with other microorganisms to manage a successful and efficient inoculation. To maximise the likelihood of successful colonisation and the subsequent establishment of an efficient symbiotic function in the roots, the method and timing of the PGPR inoculation on the soil and plant are crucial. The repetition of bacterial inoculations needs to be done to ensure the successful colonisation of PGPR over the competition with other microbes in the field.

Biofertiliser is usually applied to the soil and plant in two-week intervals or when necessary. It is because, a promising strategy to preserve the advantageous effects of microbial inoculants on plants and soil is repeated inoculation (Wang *et al*. 2021). The study on soybeans revealed that the effective population rate of PGPR strains was approximately 10^7^ CFU/mL–10^9^ CFU/mL (Bai *et al*. 2002). This suggests that the rate of inoculation also played a role in enhancing subsequent colonisation. Meanwhile, a population size of around 10^5^ CFU/mL–10^6^ CFU/mL was the optimum rate for wheat plants (Bashan 1986). This study showed that bacterial count in the soil was increased with the repetition inoculation of PGPR compared to control. According to Marschner *et al*. (2003), high and long-term fertilisation affects the soil microbial community structure. The root colonisation efficiency of PGPR was closely associated with microbial competition and survival in the soil. Plant roots react to different environmental conditions through the secretion of a wide range of compounds which interfere with the plant-bacteria interaction, being considered an important factor in the efficiency of the inoculants (Cai *et al*. 2012).

This study also showed that the soil properties of inoculated PGPR particularly the percentage of C and S, the availability of N, P, K, the soil pH and CEC had an improvement over the control. Soil carbon improves soil quality, functionality and health because higher carbon improves the soil’s biological (microbial biomass), chemical and physical properties (Shelake *et al.* 2019). Many studies showed that increasing soil organic carbon also improves crop growth and yield. Meanwhile, sulphur is another secondary nutrient that plays an important role in protein synthesis for plant growth. It is assumed that, in temperate climates, organic matter contains over 95% of all soil S due to a slow rate of decomposition, the profile of organic S concentration typically mirrors the pattern of organic matter concentration (Rai *et al.* 2020). These situations demonstrated the effectiveness of PGPR inoculation, as it carries PGP traits that contribute to the improvement of soil chemical properties and fertility. The slight increase in soil pH and CEC in this study might be due to the accumulation of mineral ions by the PGPR activities.

## CONCLUSION

Beneficial effects of the isolated BRIS soil bacterial inoculations on the okra planted in a polybag pot under an open field condition have been successfully demonstrated. Inoculation of UA 1 (*Paraburkholderia unamae*), UA 6 (*Bacillus amyloliquefaciens*) and UAA 2 (*Enterobacter asburiae*) either in single or in consortium with the addition of organic material and 70% NPK fertiliser have successfully promoted okra growth, physiology, yield and soil physicochemical properties as compared to the untreated control with 100% NPK fertiliser. This study has successfully demonstrated that the use of BRIS soil PGPR could reduce 30% of chemical fertiliser in planting crops. These situations could be attributed to the effective PGPR inoculations which have a great potential to be used as bioinoculants or in biofertiliser formulations to increase plant growth and yield and soil fertility for more sustainable agriculture practice.

## Figures and Tables

**Table 1:** The effect of BRIS soil PGPR addition on growth parameters of okra grown for 78 days.

Treatment	Number of leaf	Leaf area (cm^2^)	Stem diameter (cm)	Plant height (cm)
T1	30.31 ± 1.35^a^	692.20 ± 8.23^a^	9.16 ± 0.93^a^	150.81 ± 8.98^a^
T2	35.13 ± 3.62^b^	710.05 ± 9.73^b^	9.21 ± 0.64^a^	155.18 ± 8.49^a^
T3	36.44 ± 2.87^bc^	712.81 ± 4.59^b^	9.20 ± 0.56^a^	154.05 ± 9.77^a^
T4	34.75 ± 1.79^b^	708.52 ± 2.90^b^	9.29 ± 0.59^a^	154.16 ± 11.24^a^
T5	38.75 ± 2.62^c^	711.40 ± 8.43^b^	9.21 ± 0.63^a^	154.28 ± 8.98^a^

*Notes.* Mean ± standard deviation with the same letters in the same column are not significantly different at *P* < 0.05 Tukey’s multiple comparison. 100% of NPK fertiliser, (T1); 70% of NPK fertiliser with addition of single strain of UA 1 (T2), UA 6 (T3), UAA 2 (T4) and mix strains of UA 1, UA 6 and UAA 2 (T5).

**Table 2:** The effect of BRIS soil PGPR addition on okra physiology grown for 78 days.

Treatment	Net photosynthesis (μmol/m^2^/sec)	Stomata conductance (mol/m^2^/sec)	Internal sub-stomata CO_2_ (mol/m^2^/sec)	Transpiration rate (E_1_) (μmol/m^2^/sec)	Leaf to air vapour pressure deficit (kPa)
T1	16.70 ± 2.27^a^	1.40 ± 0.23^a^	301.25 ± 12.56^a^	6.37 ± 1.66^a^	0.77 ± 0.10^a^
T2	17.94 ± 3.02^a^	1.86 ± 0.47^b^	306.88 ± 7.46^a^	8.30 ± 0.48^b^	0.72 ± 0.11^a^
T3	17.46 ± 1.79^a^	1.70 ± 0.23^ab^	305.75 ± 8.29^a^	7.80 ± 0.58^b^	0.74 ± 0.25^a^
T4	17.30 ± 1.48^a^	1.56 ± 0.38^ab^	303.69 ± 7.71^a^	7.43 ± 1.11^b^	0.75 ± 0.06^a^
T5	17.58 ± 2.26^a^	1.75 ± 0.43^ab^	307.50 ± 5.45^a^	7.97 ± 0.44^b^	0.73 ± 0.07^a^

*Notes.* Mean ± standard deviation with the same letters in the same column are not significantly different at *P* < 0.05 Tukey’s multiple comparison. 100% of NPK fertiliser, (T1); 70% of NPK fertiliser with addition of single strain of UA 1 (T2), UA 6 (T3), UAA 2 (T4) and mix strains of UA 1, UA 6 and UAA 2 (T5).

**Table 3:** Dry weight of plant parts and total dry biomass (g) of okra grown with BRIS soil PGPR for 78 days.

Treatment	Dry weight of plant part (g)			Total dry biomass (g)

	Leaf	Stem	Root	
T1	33.75 ± 2.84^a^	86.00 ± 4.38^a^	53.50 ± 3.38^a^	173.25 ± 8.34^a^
T2	37.25 ± 3.97^a^	100.75 ± 7.20^a^	70.00 ± 4.66^bc^	208.00 ± 8.57^bc^
T3	42.50 ±3.97^a^	97.75 ± 2.56^a^	66.75 ± 3.73^abc^	207.00 ± 4.36^bc^
T4	36.00 ± 2.48^a^	90.25 ± 1.84^a^	58.50 ± 3.12^ab^	184.75 ± 3.09^ab^
T5	43.25 ± 1.93^a^	100.50 ± 3.52^a^	74.25 ± 1.55^c^	218.00 ± 4.88^c^

*Notes.* The results are presented as the mean of okra dry biomass ± standard error. Mean with the same letters in the same column are not significantly different at *P* < 0.05 Tukey’s multiple comparison. 100% of NPK fertiliser, (T1); 70% of NPK fertiliser with addition of single strain of UA 1 (T2), UA 6 (T3), UAA 2 (T4) and mix strains of UA 1, UA 6 and UAA 2 (T5).

**Table 4:** Chemical properties and total bacterial count of soil grown with okra and BRIS soil PGPR for 78 days.

Treatment	Carbon (%)	Sulfur (%)	Nitrogen (mg/kg)	Phosphorus (mg/kg)	Potassium (mg/kg)	pH	CEC (Cmol_c_/kg)	Bacteria count (Log_10_ cells/g soil)
T1	2.321± 0.004^a^	0.034± 0.001^ab^	45.147± 0.733^a^	21.710± 0.944^a^	31.777± 1.073^a^	4.613± 0.007^a^	5.073± 0.811^a^	9.610 ± 0.019^a^
T2	2.561± 0.022^c^	0.045± 0.002^b^	56.263± 1.569^c^	27.870± 0.841^c^	37.863± 0.783^b^	4.803± 0.003^c^	7.123± 0.286^c^	11.452 ± 0.015^c^
T3	2.765± 0.003^d^	0.035± 0.002^ab^	58.707± 0.485^c^	23.757± 0.485^ab^	35.993± 0.134^b^	4.707± 0.006^b^	5.997± 0.109^b^	11.710 ± 0.014^d^
T4	2.433 ±0.010^b^	0.033± 0.002^a^	50.280± 0.935^b^	24.747± 0.641^abc^	35.617± 0.345^b^	4.697± 0.018^b^	5.837± 0.041^b^	11.253 ± 0.031^b^
T5	2.818 ± 0.008^e^	0.035± 0.004^ab^	57.323± 0.615^c^	25.933± 0.525^bc^	36.570± 0.800^b^	4.807± 0.003^c^	7.047± 0.154^c^	11.800 ± 0.010^d^

*Notes.* The results show as mean of okra soil chemical properties and total bacterial count ± standard error. Means with the same letters in the same column are not significantly different at *P* < 0.05 Tukey’s multiple comparison. 100% of NPK fertiliser, (T1); 70% of NPK fertiliser with addition of single strain of UA 1 (T2), UA 6 (T3), UAA 2 (T4) and mix strains of UA 1, UA 6 and UAA 2 (T5).
